# Dietary supplementation with whey protein improves systemic microvascular function in heart failure patients: a pilot study

**DOI:** 10.1590/1414-431X202010577

**Published:** 2021-04-19

**Authors:** A. De Lorenzo, E.M. Dos Santos, A.S. Bello Moreira, G.V.B. Huguenin, E. Tibirica

**Affiliations:** 1 Instituto Nacional de Cardiologia, Rio de Janeiro, RJ, Brasil; 2 Departamento de Nutrição e Dietética, Universidade Federal Fluminense, Niterói, RJ, Brasil; 3 Departamento de Nutrição Social, Instituto de Nutrição, Universidade Estadual do Rio de Janeiro, Rio de Janeiro, RJ, Brasil

**Keywords:** Laser speckle contrast imaging, Microvascular reactivity, Endothelial function, Dietary supplementation, Skin iontophoresis

## Abstract

Endothelial dysfunction is a well-known component of the pathophysiology of heart failure (HF), with proven prognostic value. Dietary supplementation with whey protein (WP) has been widely used to increase skeletal muscle mass, but it also has vascular effects, which are less understood. This study aimed to evaluate the effects of WP supplementation on the systemic microvascular function of HF patients. This was a blinded, randomized, placebo-controlled clinical trial that evaluated the effects of 12-week WP dietary supplementation on systemic microvascular function, in patients with HF New York Heart Association (NYHA) classes I/II. Cutaneous microvascular flow and reactivity were assessed using laser speckle contrast imaging, coupled with pharmacological local vasodilator stimuli. Fifteen patients (aged 64.5±6.2 years, 11 males) received WP supplementation and ten patients (aged 68.2±8.8 years, 8 males) received placebo (maltodextrin). The increase in endothelial-dependent microvascular vasodilation, induced by skin iontophoresis of acetylcholine, was improved after WP (P=0.03) but not placebo (P=0.37) supplementation. Moreover, endothelial-independent microvascular vasodilation induced by skin iontophoresis of sodium nitroprusside, was also enhanced after WP (P=0.04) but not placebo (P=0.42) supplementation. The results suggested that dietary supplementation with WP improved systemic microvascular function in patients with HF.

## Introduction

Dietary supplementation with whey protein (WP) is known to improve blood pressure, systemic vascular resistance and arterial stiffness in overweight and obese individuals (1,2).

Endothelial dysfunction is a known component of the pathophysiology of heart failure (HF) (3,4) and has been reported to adversely affect the prognosis in several cardiovascular disorders (5). Indeed, patients with HF have chronic systemic vasoconstriction associated with reduced tissue perfusion, and endothelial dysfunction is importantly involved in this phenomenon (3,4). Adult patients with heart failure, in New York Heart Association (NYHA) functional classes II-III, and with more severe endothelial dysfunction have a higher incidence of hospitalization due to decompensation of HF, cardiac transplantation, or cardiac death in a 1-year follow-up than those with relatively preserved endothelium-dependent relaxation (6). Moreover, HF results in substantial loss of well-being and financial costs worldwide, including Brazil, and is considered to be a public health priority (7).

Endothelial microvascular function may currently be evaluated using laser speckle contrast imaging (LSCI), coupled with skin micro-iontophoresis of acetylcholine (ACh) for the study of endothelial-dependent vasodilation, and sodium nitroprusside (SNP) for endothelium-independent vasodilation. This method allows measurements of microvascular blood flow with very good reproducibility (8) and has been employed to evaluate microvascular function in several contexts (9-11).

Nonetheless, the vascular effects of WP supplementation are still not fully understood and, especially in HF patients, the effects are unknown. Thus, this study aimed to determine the effects of a 12-week dietary WP supplementation, compared to placebo, on microvascular function, evaluated using LSCI in patients with HF.

## Material and Methods

This study was approved by the Institutional Review Board of the National Institute of Cardiology (CAAE #03218512.0.2005.5272) and registered and published on clinicaltrials.gov (NCT03142399). Written informed consent was obtained from all patients before inclusion in the study, which was carried out between July 2018 and March 2019 at the Clinical Research Department of the National Institute of Cardiology, Ministry of Health, Brazil.

**Figure 1:**
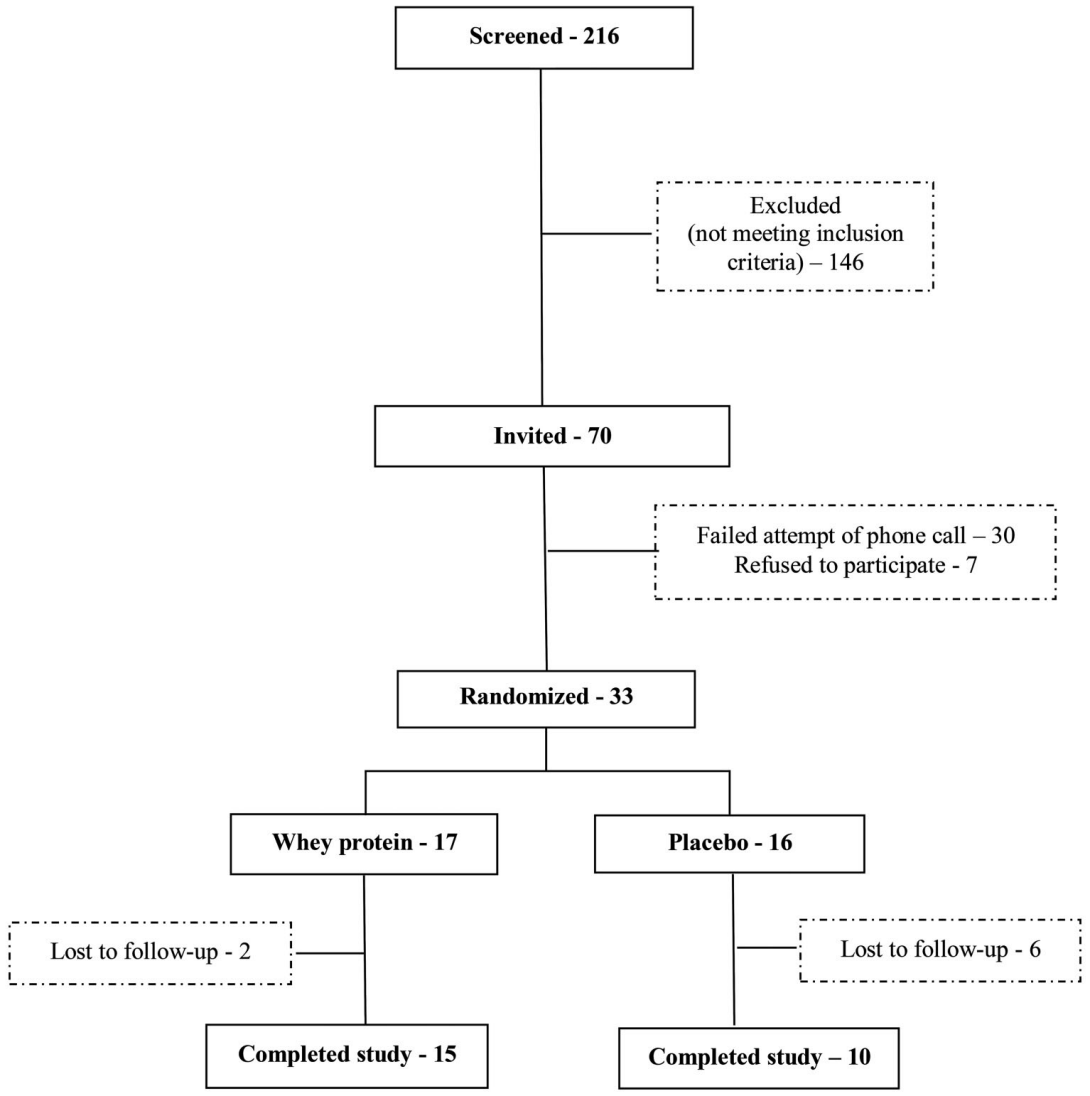
Flow chart of the selection of study population.

Initially, 216 patients with HF were screened for participation in the study (Figure 1); 146 patients were excluded because they did not meet inclusion criteria for the study. After selection, 70 patients were considered eligible for the study, but 37 were not found (failed contact attempt by phone call) or refused to participate. Thirty-three patients were randomized to the study, but eight were lost to follow-up, and 25 patients completed the study (Figure 1).This was a blinded, randomized, placebo-controlled clinical trial in which participants were randomly allocated into two groups to receive supplementation: WP (30 g per day, totaling 27 g of protein and 120 kcal per serving; Isowhey, Max titanium, Supley Laboratórios de Alimentos e Suplementos Nutricionais Ltda., Brazil), the product was also composed of 1.5 g of maltodextrin and 1.5 g of vitamins and minerals; or placebo (30 g maltodextrin per day, totaling 30 g of carbohydrates and 120 kcal per serving). Maltodextrin was chosen as an appropriate control because it was flavorless and easily dissolved and had an energy content that matched that of the milk proteins. This placebo has been used previously to study the effects of WP on vascular endothelial function (1).

Randomization was based on a table of random numbers (generated in OpenEpi^®^, <http://openepi.com>) that were blinded from all researchers, except for one who encoded the bottles of supplements and had no contact with the center at which the study was conducted. Outcomes were measured at baseline and upon completion of 12 weeks of study; therefore, the microvascular reactivity tests were performed twice: the first time at baseline, before the initiation of either WP or placebo supplementation, and the second time after 12 weeks of supplementation. All participants underwent all microcirculatory examinations.

Further details of the study protocol may be found elsewhere (12). Briefly, inclusion criteria were ≥50 years of age, HF class I or II NYHA with stable symptoms for at least 4 weeks prior to enrollment, and left ventricular ejection fraction ≤50%. Exclusion criteria were reduced creatinine clearance, allergies to milk proteins, or inability to understand and perform the study procedures (12). The packaging and distribution of the supplements (delivered to the patients in metalized packages, sealed and unidentifiable as placebo or WP) was performed by personnel not involved in the research.

The microcirculatory tests were performed in a room with stable temperature (approximately 23°C) after a 20-min rest period in the supine position. Microvascular flow and reactivity were evaluated using a laser speckle contrast imaging (LSCI) system with a laser wavelength of 785 nm (PeriCam PSI system, Perimed, Sweden) in combination with the iontophoresis of acetylcholine (ACh) or sodium nitroprusside (SNP) in arbitrary perfusion units (APU). Two drug-delivery electrodes were installed on the ventral surface of the forearm by means of adhesive discs (LI 611, Perimed). ACh 2% w/v or SNP 2% w/v (Sigma Chemical Co., USA) iontophoresis was performed using a micropharmacology system (PF 751 PeriIont USB Power Supply, Perimed) with increasing anodal (ACh) or cathodal (SNP) currents of 30, 60, 90, 120, 150, and 180 μA for 10-s intervals spaced 1 min apart. Skin blood flow measurements in APU were divided by mean arterial pressure values to yield cutaneous vascular conductance (CVC) in APU/mmHg. The results of the pharmacological tests are reported as peak values, representing the maximal microvascular vasodilation observed after the highest dose of ACh or SNP.

The data are reported as means±SD. Variables without a Gaussian distribution (Shapiro-Wilk normality test) are reported as the median (25th-75th percentiles). Comparisons of parameters obtained before and after treatment with placebo or WP were performed using two-way ANOVA followed by multiple comparisons (Sidak's multiple comparisons test), considering the interactions of time (pre- and post-treatment) and treatment (placebo or WP). Baseline characteristics of patients were analyzed by two-sided unpaired Student's *t*-test, chi-squared test, or Fisher's exact test. The statistical package used was Prism version 7.0 (GraphPad Software Inc., USA).

## Results

Table 1 shows the clinical characteristics of the placebo and WP supplementation groups. There were no significant differences in baseline parameters between groups.

The baseline values of microvascular flow showed a tendency to increase after both placebo and WP supplementation; nevertheless, these differences did not reach statistical significance (Table 2). Microvascular flow was 0.24 (0.17-0.32) APU/mmHg before and 0.32 (0.29-0.42) APU/mmHg after supplementation with placebo (P=0.05). After WP supplementation, microvascular flow was 0.25 (0.22-0.32) APU/mmHg before and 0.33 (0.24-0.41) APU/mmHg after supplementation (P=0.09). The peak values of CVC during skin iontophoresis of ACh before placebo supplementation [0.36 (0.29-0.55) APU/mmHg] did not change significantly after the 12-week period [0.45 (0.31-0.67) APU/mmHg] (P=0.37). In contrast, there was a significant increase of CVC after WP supplementation: 0.39 (0.33-0.51) APU/mmHg before and 0.54 (0.36-0.68) APU/mmHg after 12 weeks (P=0.03). Skin iontophoresis of SNP resulted in a similar pattern of response. The peak values of CVC during skin iontophoresis of SNP before placebo supplementation [0.35 (0.29-0.43) APU/mmHg] did not change significantly after the 12-week period [0.44 (0.33-0.50) APU/mmHg] (P=0.42). In contrast, there was a significant increase of CVC after WP supplementation: 0.35 (0.29-0.41) APU/mmHg before and 0.50 (0.34-0.71) APU/mmHg after 12 weeks (P=0.04). These data are shown in Table 2.

**Table 1:** Clinical characteristics of patients included in the groups of dietary supplementations with whey protein or placebo (12 weeks).

Parameters	Whey protein (n=15)	Placebo (n=10)	P values
Age (years)	64.5±6.2	68.2±8.8	0.23
Male, n (%)	11 (73.3)	8 (80)	0.07
BMI (kg/m^2^)	28.6±4.9	26.1±3.4	0.18
SAP (mmHg)	144.1±20.5	149.1±26.7	0.58
DAP (mmHg)	87.5±26.4	82.4±8.3	0.56
LVEF (%, Teicholz)	42.5±6.7	40.9±8.4	0.64
NYHA class I, n (%)	13 (86.7)	6 (60)	0.12
Type 2 diabetes, n (%)	7 (46.7)	4 (40)	0.10
Dyslipidemia, n (%)	11 (73.3)	7 (70)	0.85
Arterial hypertension, n (%)	12 (80)	7 (70)	0.32
AMI, n (%)	15 (100)	10 (100)	>0.99
MR/PCI, n (%)	14 (93.3)	9 (90)	0.76
Smoker/ex-smoker, n (%)	13 (86.7)	7 (70)	0.31

The data are reported as means±SD or absolute numbers and percentages. Comparisons of numerical variables were analyzed by two-sided unpaired Student *t*-tests and categorical variables were analyzed by chi-squared or Fisher's exact test. BMI: body mass index; SAP: systolic arterial pressure; DAP: diastolic arterial pressure; LVEF: left ventricular ejection fraction; NYHA: New York Heart Association; AMI: acute myocardial infarction; MR: myocardial revascularization; PCI: percutaneous coronary intervention.

**Table 2:** Effects of 12-week dietary supplementation with placebo or whey protein on systemic microvascular flow and reactivity of patients with chronic heart failure.

Parameter (APU/mmHg)	Placebo			Whey protein		
	Pre	Post	P value	Pre	Post	P value
Baseline CVC	0.24 (0.17-0.32)	0.32 (0.29-0.42)	0.05	0.25 (0.22-0.32)	0.33 (0.24-0.41)	0.09
Peak values CVC (Ach)	0.36 (0.29-0.55)	0.45 (0.31-0.67)	0.37	0.39 (0.33-0.51)	0.54 (0.36-0.68)	**0.03**
Peak values CVC (SNP)	0.35 (0.29-0.43)	0.44 (0.33-0.50)	0.42	0.35 (0.29-0.41)	0.50 (0.34-0.71)	**0.04**

APU: arbitrary perfusion units; Pre: before supplementation; Post: after supplementation; CVC: cutaneous vascular conductance; Ach: acetylcholine; SNP: sodium nitroprusside. Data are reported as medians (25th-75th percentiles). Results were analyzed using two-way ANOVA followed by multiple comparisons (Sidak's multiple comparisons test), where we considered the interactions of time (pre- and post-treatment) and dietary supplementation (placebo or whey protein). P values in bold denote statistically significant effects (P<0.05).

## Discussion

In this pilot study, an increase of endothelial-dependent and endothelial-independent vasodilation was found after WP supplementation for 12 weeks. The endothelial-dependent vasodilation may be linked to the effects of arginine, a nitric oxide precursor that is found in significant amounts in WP (13). In fact, L-arginine is the substrate for the enzyme endothelial nitric oxide synthase, which is responsible for the endothelial production of nitric oxide (13). Previous studies have shown increased flow-mediated vasodilation with WP or WP-derivatives in healthy adults (14), in patients with cardiovascular risk factors (15), and in patients with mild hypertension (1); however, no study had yet assessed systemic microcirculation by LSCI.

It is important to note that macro- and microvascular dysfunctions are defined as the impairment of endothelium-dependent vasodilation and/or increased arterial stiffness, which are related to several cardiovascular diseases, including hypertension, atherosclerosis, diabetes, and heart failure (16). The study of Fekete et al. (1), for instance, showed that the consumption of WP for eight weeks resulted in clinically relevant reductions in blood pressure, using ambulatory blood pressure measurements, in adults with hypertension compared with controls. Moreover, it has recently been shown that endothelium-dependent vasodilation, evaluated by brachial artery flow-mediated dilatation, is improved after acute supplementation with WP hydrolysates (single-dose administration of 20 g of WP) in healthy volunteers (14). In another randomized cross-over study carried out in older individuals with impaired endothelial function (low brachial artery flow-mediated dilatation), acute ingestion of an extract derived from WP improved endothelium-dependent vasodilation (15).

Regarding endothelial-independent vasodilation, possible mechanisms are less clear; it is known that WP modulates premature senescence of vascular smooth muscle cells triggered by angiotensin II through transcriptional up-regulation of SIRT1 expression, what may provide evidence of improved vascular homeostasis (17). Additionally, it has been demonstrated, both in endothelial and in smooth muscle cells, that WP peptides inhibit ACE (18). The WP peptide also decreases the expression of pro-matrix metalloproteinase 2 (MMP2) and, consequently, the activity of MMP2, and might reduce aortic stiffness occurring in pathological conditions, besides aging (19). Thus, supplementation with WP improves arterial stiffness, together with reduction of both systolic and diastolic arterial pressure in overweight/obese individuals (2).

### Conclusions

Our results suggested that dietary supplementation with WP improved microvascular endothelial function and possibly smooth muscle structure in patients with HF. This pilot study was limited by the small number of participants, which may preclude the extrapolation of its findings to larger populations of patients with HF. Nevertheless, it is worth mentioning that the improvement in microvascular function was observed after long-term supplementation with WP, controlled with placebo, which is an original finding in the specialized literature. Moreover, it showed new findings regarding the vascular effects of WP, which may turn this nutrient into another constituent of therapeutic regimen of patients with HF in the future and after larger trials.
